# Isolation and Functional Characterization of the Novel *Clostridium botulinum* Neurotoxin A8 Subtype

**DOI:** 10.1371/journal.pone.0116381

**Published:** 2015-02-06

**Authors:** Skadi Kull, K. Melanie Schulz, Jasmin Weisemann née Strotmeier, Sebastian Kirchner, Tanja Schreiber, Alexander Bollenbach, P. Wojtek Dabrowski, Andreas Nitsche, Suzanne R. Kalb, Martin B. Dorner, John R. Barr, Andreas Rummel, Brigitte G. Dorner

**Affiliations:** 1 Biological Toxins (ZBS3), Centre for Biological Threats and Special Pathogens, Robert Koch-Institut, Berlin, Germany; 2 Institut für Toxikologie, Medizinische Hochschule Hannover, Hannover, Germany; 3 Highly Pathogenic Viruses (ZBS1), Centre for Biological Threats and Special Pathogens, Robert Koch-Institut, Berlin, Germany; 4 Centers for Disease Control and Prevention, National Center for Environmental Health, Division of Laboratory Sciences, Atlanta, Georgia, United States of America; Institute Pasteur, FRANCE

## Abstract

Botulism is a severe neurological disease caused by the complex family of botulinum neurotoxins (BoNT). Based on the different serotypes known today, a classification of serotype variants termed subtypes has been proposed according to sequence diversity and immunological properties. However, the relevance of BoNT subtypes is currently not well understood. Here we describe the isolation of a novel *Clostridium botulinum* strain from a food-borne botulism outbreak near Chemnitz, Germany. Comparison of its botulinum neurotoxin gene sequence with published sequences identified it to be a novel subtype within the BoNT/A serotype designated BoNT/A8. The neurotoxin gene is located within an *ha-orfX+* cluster and showed highest homology to BoNT/A1, A2, A5, and A6. Unexpectedly, we found an arginine insertion located in the HC domain of the heavy chain, which is unique compared to all other BoNT/A subtypes known so far. Functional characterization revealed that the binding characteristics to its main neuronal protein receptor SV2C seemed unaffected, whereas binding to membrane-incorporated gangliosides was reduced in comparison to BoNT/A1. Moreover, we found significantly lower enzymatic activity of the natural, full-length neurotoxin and the recombinant light chain of BoNT/A8 compared to BoNT/A1 in different endopeptidase assays. Both reduced ganglioside binding and enzymatic activity may contribute to the considerably lower biological activity of BoNT/A8 as measured in a mouse phrenic nerve hemidiaphragm assay. Despite its reduced activity the novel BoNT/A8 subtype caused severe botulism in a 63-year-old male. To our knowledge, this is the first description and a comprehensive characterization of a novel BoNT/A subtype which combines genetic information on the neurotoxin gene cluster with an in-depth functional analysis using different technical approaches. Our results show that subtyping of BoNT is highly relevant and that understanding of the detailed toxin function might pave the way for the development of novel therapeutics and tailor-made antitoxins.

## Introduction

Botulism, a life-threatening disease in humans and animals, is caused by botulinum neurotoxins (BoNTs) that are produced by the Gram-positive, anaerobic, spore-forming bacterium *Clostridium (C.) botulinum* together with non-toxic associating proteins. To date there are seven confirmed BoNT serotypes (A–G), and a proposed one (H), of which BoNT/A, B, E, F and the proposed H are associated with botulism in humans, while BoNT/C and D cause disease primarily in animals. BoNT/G has not yet been clearly linked to a botulism outbreak in humans or animals [1–5]. Recently, detailed genetic and proteomic comparisons revealed that most serotypes can be divided into several subtypes. Smith *et al*. defined subtypes as two BoNTs of the same serotype that differ by at least 2.6% in amino acid sequence [6]. However, BoNT variants showing higher identity have been reported as distinct subtypes for BoNT/B and E [7,8]. With respect to BoNT/A, five subtypes BoNT/A1 to A5 have been described [7]. Two additional subtypes have been described which fit the above-mentioned definition, but have not been explicitly designated as new subtypes by the authors [9,10]. Within this paper, we refer to these molecules as BoNT/A6 and A7, respectively. Altogether, 32 subtypes have been identified so far for the serotypes relevant to humans (BoNT/A1 to A7, B1 to B8, E1 to E11, F1 to F7) [6–9,11–17]. For BoNT/C and D no subtypes but mosaic forms have been reported [15,18].

The *bont* gene is part of a gene cluster that has been shown to exist in two different forms: *ha* ^+^ *orfX* ^-^cluster or *ha* ^-^ *orfX* ^+^ cluster that differ in the composition and arrangement of genes [1,19]. All but serotype A are associated with a single cluster type. In serotype A the subtypes *bont/a1* and *a5* are located within the *ha* ^+^ *orfX* ^-^cluster; the genes for *bont/a1*, *a2*, *a3*, *a4*, and *a6* are found within the *ha* ^-^ *orfX* ^+^ cluster. Thus, so far only the *bont/a1* subtype exists in both clusters.

All botulinum neurotoxins are synthesized as single molecules of 150 kDa. Upon activation by a protease they form dichain toxins composed of a 50 kDa light chain (LC) with zinc-dependent protease activity, linked via a disulfide bond to the 100 kDa heavy chain, comprising a *N*-terminal translocation domain (H_N_) and a *C*-terminal cell binding domain (H_C_) [20–22].

After reaching the neuromuscular junction, BoNT first accumulate via binding to complex polysialo-gangliosides on the surface of motoneurons and subsequently endocytose via interaction with protein receptors into small synaptic vesicles [23,24]. BoNT/A displays a single ganglioside binding site in its H_C_ fragment [25,26] and employs the three isoforms of the synaptic vesicle glycoprotein 2 (SV2A–C) as protein receptor [27,28]. Our analysis shows that the ganglioside binding site motif E…H…SXWY…G is conserved throughout BoNT/A1–A8, but both ganglioside and protein receptor binding of BoNT/A subtypes have not yet been investigated systematically.

Depending on the serotype, BoNT hydrolyze different neuronal substrate molecules. While BoNT/A [29,30], C [31] and E [30] cleave synaptosomal-associated protein of 25 kDa (SNAP-25), BoNT/B [21], D [32], F [33], and G [34] cleave synaptobrevin-2/vesicle-associated membrane protein-2 (VAMP-2) at individual peptide bonds.

Currently the overall relevance of BoNT subtypes is not yet understood. Only few publications address the characteristics and differences of botulinum neurotoxin subtypes in detail. It is known that different subtypes of a given serotype can display different binding characteristics to monoclonal and polyclonal antibodies [6,35–39].

Regarding their functional activity, only little information is available. For the four subtypes BoNT/A1–A4 Henkel *et al*. showed that their isolated recombinant LC bind SNAP-25 with similar affinity but have different turnover frequencies (k_cat_) for SNAP-25 cleavage [40]. In another study, differential catalytic properties of BoNT/A1 and A5 were described [41]. However, a recent study showed how different subtypes of a single serotype might behave: it was shown that the subtype BoNT/F5 cleaves synaptobrevin-2 at a different peptide bond than all other BoNT/F subtypes, this being the first example of a different substrate cleavage site among subtypes of a given serotype [42]. While this behavior might well be an exception among the known subtypes of BoNT serotypes, understanding functional differences is of crucial importance both for detection technology and for the development of BoNT-based therapeutics and countermeasures.

With respect to detection, most methods currently employed are based on the detection of part of the sequence (e.g. PCR, DNA arrays), on the specific binding of antibodies (e.g. ELISA, immuno PCR) or on the detection of BoNT functional activity (e.g. mouse bioassay, mouse phrenic nerve hemidiaphragm assay [MPN assay] or endopeptidase assays) or combinations thereof [43]. Besides identifying novel BoNT subtypes, this implicates that a detailed characterization and analysis of subtypes known so far and especially of novel ones is essential to ensure BoNT detection and to improve BoNT detection methods in order to be able to detect all BoNT subtypes.

With respect to therapy, the identification and functional characterization of BoNT subtypes holds the promise of defining improved or alternative therapeutics with modified efficacy or target spectrum.

The present study describes the isolation, identification and characterization of a novel botulinum neurotoxin subtype designated BoNT/A8. The novel strain was isolated from green bean salad causing food-borne botulism in Germany. BoNT/A8 was further characterized by genomic analysis and mass spectrometric protein sequencing. Additionally, the functional activity of the novel BoNT/A8 subtype was comprehensively investigated by addressing the catalytic activity, the receptor binding and the neurotoxicity of the molecule.

## Results

### Isolation of a BoNT/A8 producing strain

During a food-borne botulism outbreak affecting a 63-year-old male near the city of Chemnitz in December 2007, suspected food samples (salad of home-canned green beans and two different types of sausages) were investigated. The green beans were found positive by multiplex qPCR for *bont/a* prior and after anaerobic enrichment culture [44]. Subsequently, the *C*. *botulinum* strain “Chemnitz” was isolated.

### Genomic characterization

Sequencing of the 16S rRNA and *bont/a* genes of strain Chemnitz confirmed the identity of a BoNT/A producing *C*. *botulinum* group I strain and revealed that the *bont/a* gene sequence differed from those published by more than 2.6%, indicating that it might be a novel subtype (Table 1). The novel *bont/a* gene showed highest identity to *bont/a1*, *a5* and *a6* (96.2–96.6%). On protein level, the translated sequence differed by at least 6.6% from BoNT/A2 and A5 (Table 1). Most distant from all currently known BoNT/A subtypes was BoNT/A3 with 6.9% and 12.3% difference on nucleotide and amino acid level, respectively. The novel BoNT/A subtype of strain Chemnitz was designated BoNT/A8, the next consecutive subtype number.

**Table 1:** Identity of BoNT/A8 to other BoNT/A subtypes on nucleic acid and amino acid levels.*

		% pairwise identity											
A8 Chemnitz		LC		H_N_		H_CN_		H_CC_		H_C_		holotoxin	
sub-type	strain	*nt*	aa	nt	aa	nt	aa	nt	aa	nt	aa	nt	aa
A1	ATCC3502	**97.9**	**96.8**	**97.7**	**95.4**	*91.0*	*83.0*	97.1	92.6	*93.9*	*87.6*	**96.5**	**93.3**
A1(B)	NCTC2916	**97.8**	**96.3**	**97.7**	**95.4**	*91.0*	*83.0*	**97.1**	92.6	*93.9*	*87.6*	**96.5**	**93.1**
A2	Kyoto F	**97.8**	**96.8**	93.5	90.0	**95.7**	**92.4**	**97.6**	**94.6**	**96.6**	**93.4**	96.0	**93.4**
A3	Loch Maree	*90.5*	*82.4*	*92.7*	*88.2*	**95.4**	**91.9**	**97.4**	93.6	**96.3**	92.7	*93.1*	*87.7*
A4	657Ba	93.8	87.9	*93.1*	89.4	94.8	88.8	96.6	91.2	95.6	89.9	94.2	89.1
A5	H04402065	**98.0**	**96.3**	97.0	94.2	92.7	86.5	96.9	93.1	94.7	89.7	**96.6**	**93.4**
A6	CDC41370	**97.8**	**96.6**	**97.2**	**95.1**	92.5	85.7	*94.8*	*88.7*	*93.6*	*87.1*	**96.2**	**93.0**
A7	2008–148	**97.5**	95.0	94.4	89.4	93.0	87.0	96.1	92.2	94.5	89.5	95.5	91.3
E3	Alaska E43	49.5	32.7	57.4	36.9	70.8	52.4	56.6	37.1	64.0	45.1	56.9	38.1
F1	Langeland	52.3	32.7	56.6	36.9	72.6	54.9	59.6	42.2	66.4	48.8	58.4	39.4

* Shown is the percentage of nucleic acid (nt) and amino acid (aa) identity among the subtypes BoNT/A1 to A8 subdivided into light chain (LC), *N*-terminal domain (H_N_) and *C*-terminal domain (H_C_) of the heavy chain (HC) and of the holotoxin. Additionally, the *N*-terminal (H_CN_) and *C*-terminal (H_CC_) subdomains of H_C_ are displayed. Highest percentage of identity (±0.5) is depicted in **bold** and lowest percentage of identity (±0.5) is depicted in *italic*.

**Fig 1:**
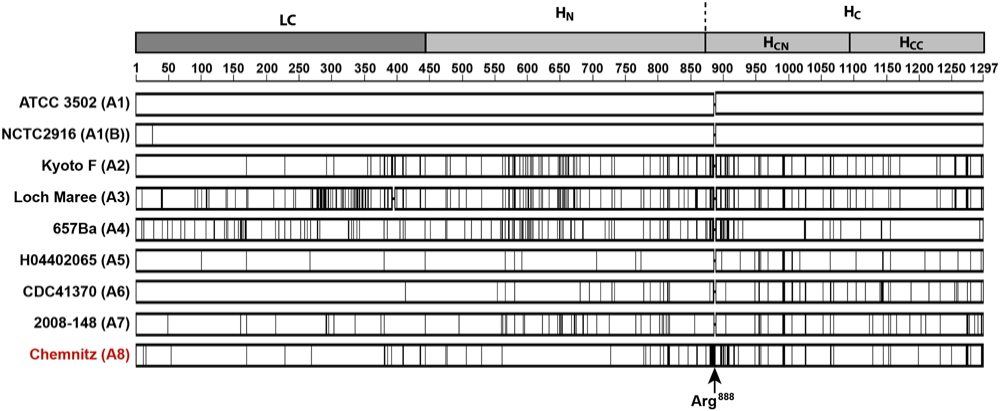
Comparison of BoNT/A subtypes. BoNT/A1 amino acid sequence of strain ATCC 3502 as prototype was compared to representatives of subtypes BoNT/A2 to A7 and the novel BoNT/A8. Sequence differences are indicated by vertical lines. The cartoon indicates the domain of the light chain (LC), the *N*-terminal part (H_N_) and the *C*-terminal part (H_C_) of the heavy chain (HC). The latter can be further subdivided into an *N*-terminal (H_CN_) and *C*-terminal (H_CC_) subdomain. The alignment illustrates that the majority of differences between BoNT/A8 and A1 are located in the heavy chain. Furthermore, the arginine insertion at position 888 is unique to BoNT/A8.

**Fig 2:**
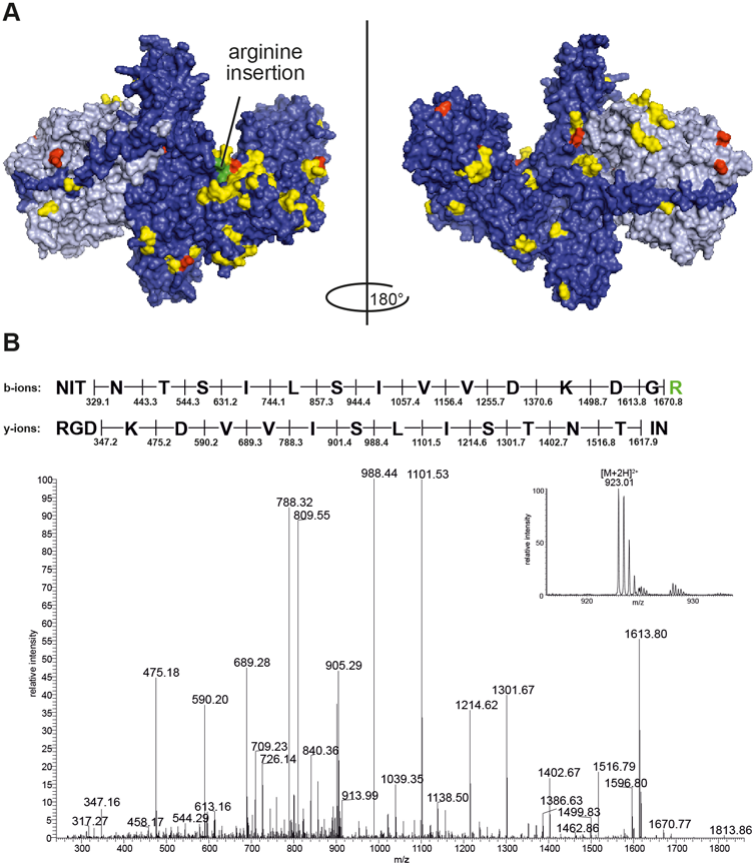
Surface representation of BoNT/A8 and arginine insertion. (A) Surface representation of BoNT/A (PDB code 3BTA [96]). Differences of BoNT/A8 to BoNT/A1 are marked in yellow, unique amino acid differences in BoNT/A8 (based on the representatives of BoNT/A2 to A7 used in Fig. 1) are marked in red, and the arginine insertion in position 888 is marked in green. (B) Representative MS/MS spectra of the precursor ion m/z 923.01 ([M+2H]^2+^], inset) of BoNT/A8 with the sequence of ^872^NITNTSILSIVVDKDGR^888^, showing the additional arginine at position 888 leading to an additional trypsin cleavage site.

Fig. 1 shows a schematic representation of BoNT/A1 to BoNT/A8, with vertical lines representing differences in amino acids compared to BoNT/A1. The alignment illustrates that most differences between BoNT/A1 and BoNT/A8 are located on the heavy chain of the molecule. A unique feature of BoNT/A8 was a triplet insertion (AGG) at position 2662, leading to an additional arginine at amino acid position 888. This makes BoNT/A8 the second 1297 aa long BoNT/A subtype next to the BoNT/A2 of strain CDC2171 [45] (Fig. 1 and S1 Fig.). Overall, BoNT/A8 showed 87 amino acid mutations compared to BoNT/A1, with 12 amino acid substitutions at positions being unique for BoNT/A8 (Fig. 2A and S1 Fig.).

We compared the identities of the four domains and subdomains of BoNT/A8 to other BoNT/A subtypes and also to BoNT/E and BoNT/F as closest relatives (Table 1). The identities for the domains and subdomains vary greatly. *E*.*g*. whereas the holotoxin and LC of BoNT/A8 had the lowest identity towards BoNT/A3 (Loch Maree), the identity of the H_CN_ subdomain between BoNT/A8 and BoNT/A3 was one of the highest. Conversely, BoNT/A1 showed the highest identity to BoNT/A8 for the LC and H_N_ domains whereas the H_CN_ subdomain differed the most.

**Fig 3:**
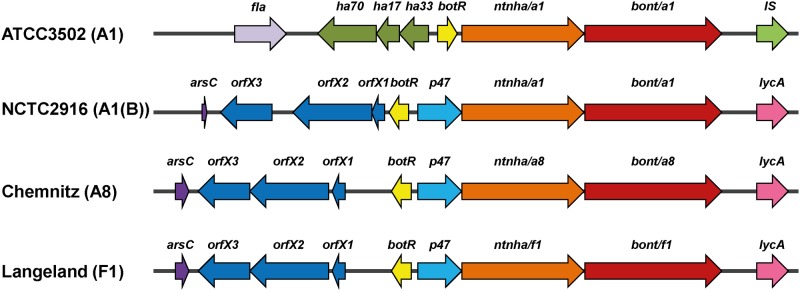
*Bont/a8* gene cluster organization. Schematic representation of the neurotoxin gene clusters from the prototype BoNT/A1 strain ATCC 3502, BoNT/A1(B) strain NCTC 2916, BoNT/A8 strain Chemnitz, and BoNT/F1 strain Langeland. The *bont/a1* gene from ATCC 3502 is located within an *ha* ^+^ *orfX* ^-^cluster containing the *non-toxic non-haemagglutinin* (*ntnha*) gene, the three *haemagglutinin* genes (*ha70*, *ha17* and *ha33*) and the regulatory *botR* gene. The *bont/a1* neurotoxin gene cluster of ATCC 3502 is flanked by the *flagellin* (*fla*) gene and an insertion sequence element (IS). In contrast to the prototype *bont/a1* (ATCC 3502), the *bont/a1* of NCTC 2916 and the *bont/a8* gene of Chemnitz are located in an *ha* ^-^ *orfX* ^+^ cluster which contains the adjacent *ntnha* gene and—instead of the *ha* genes—the three *orfX1–3* genes; apart from the *botR* gene the cluster also contains the *p47* gene of unknown function. The neurotoxin gene clusters of NCTC 2916 and Chemnitz are located within the ars operon on the chromosome and are flanked by the *arsC* and the *lycA* genes. Except for the toxin gene itself, its gene cluster organization and sequence is most similar to the *bont/f1* gene cluster of strain Langeland.

BoNT/A is the only serotype encoded either in an *ha* ^+^ *orfX* ^-^or an *ha* ^-^ *orfX* ^+^ neurotoxin gene cluster. To elucidate the organization of the neurotoxin gene cluster of strain Chemnitz, we applied whole genome shotgun sequencing (454 sequencing on a GS FLX+ System, Roche). Additionally, the gene cluster was partly verified by Sanger sequencing and is available under GenBank accession number KM233166. Noteworthy, Sanger sequencing of the *bont/a* gene with the primers published by Hill *et al*. [7] resulted in a different last amino acid as compared to the 454 shot gun sequence (L→Q). This is based on the fact that the Sanger sequence was forced by the primer 6R sequence to CTG (encoding L) instead of the CAG (encoding Q) which was unambiguously obtained by whole genome sequencing. The *bont*/*a8* cluster belonged to the *ha* ^-^ *orfX* ^+^ type (Fig. 3) which is known to encode BoNT/A1, A1(B), A2, A3, A4, and A6 subtypes as well as BoNT/E and F serotypes [15]. The *bont*/*a8* gene cluster was located within the ars operon on the chromosome like other *ha* ^-^ *orfX* ^+^ BoNT/A- and BoNT/F-producing strains of group I and is flanked by the *arsC* and the *lycA* genes [46]. Noteworthy, the whole *bont*/*a8* gene cluster—with the exception of the *bont/a8* gene itself—was closest related to the *bont*/*f1* gene cluster of strain Langeland also belonging to *C*. *botulinum* group I (Fig. 3, Table 2 and Table 3).

**Table 2:** Sequence identity of individual genes within the neurotoxin gene cluster of strain Chemnitz encoding BoNT/A8 compared to other BoNT/A, E and F sero- and subtypes located in an *ha* ^-^ *orfx* ^+^ gene cluster on nucleotide levels.*

Subtype	Strain	*bont*	*ntnha*	*p47*	*botR*	*orfX1*	*orfX2*	*orfX3*
A1	ATCC 3502	**96.5**	*86.5*	n/a	*75.6*	n/a	n/a	n/a
A1(B)	NCTC 2916	**96.5**	94.9	**99.5**	94.9	93.2	**97.9**	96.1
A2	Kyoto F	96.0	**97.2**	86,3	96.4	**95.6**	**98.2**	**96.8**
A3	Loch Maree	*93.1*	96.0	87.4	97.7	**95.1**	97.2	*94.2*
A4	657	94.2	96.5	*81.9*	**98.3**	*91.9*	*86.8*	95.6
A5	H04402 065	**96.6**	*86.5*	n/a	*76.0*	n/a	n/a	n/a
A6	CDC41370	**96.2**	**97.4**	87.4	89.8	93.2	**98.0**	95.6
A7	2008–148	95.5						
E3	Alaska E43	56.9	85.6	85.2	n/a	79.3	66.0	82.3
F1	Langeland	58.4	98.3	99.8	100.0	99.8	99.9	99.9

* The neurotoxin gene clusters encoding for BoNT, NTNHA, p47, BotR and the OrfX1, OrfX2 and OrfX3 proteins were compared between strain Chemnitz (BoNT/A8) and the other BoNT/A subtypes (A1 to A7) and percent identities are given either on nucleic acid level (A) or on amino acid level (B). For comparison the identities to BoNT/E and BoNT/F also located within an *ha* ^-^ *orfX* ^+^ neurotoxin gene cluster are displayed, too. Highest identities (±0.5) are depicted in **bold** and lowest identities (±0.5) are depicted in *italic*. Empty cells indicate that no sequence data exist while n/a denotes the absence of certain genes from the neurotoxin gene cluster.

**Table 3:** Sequence identity of individual genes within the neurotoxin gene cluster of strain Chemnitz encoding BoNT/A8 compared to other BoNT/A, E and F sero- and subtypes located in an *ha^-^orfx^+^* gene cluster on amino acid levels.*

Subtype	Strain	BoNT	NTNHA	p47	BotR	OrfX1	OrfX2	OrfX3
A1	ATCC 3502	**93.3**	*76.7*	n/a	*61.2*	n/a	n/a	n/a
A1(B)	NCTC 2916	**93.1**	92.4	**99.3**	91.0	89.4	**97.7**	95.1
A2	Kyoto F	**93.4**	**95.5**	79.1	92.1	**93.0**	**97.1**	**95.7**
A3	Loch Maree	*87.7*	94.6	79.6	95.5	**93.0**	95.7	93.5
A4	657	89.1	94.1	*73.8*	**97.2**	*87.3*	*78.2*	*94.3*
A5	H04402 065	**93.4**	*76.7*	n/a	62.9	n/a	n/a	n/a
A6	CDC41370	**93.0**	**95.9**	79.8	79.8	89.4	**97.6**	*94.5*
A7	2008–148	91.3						
E3	Alaska E43	38.1	76.0	81.0	n/a	73.9	51.3	76.7
F1	Langeland	39.4	96.2	100.0	100.0	99.3	99.7	99.6

* The neurotoxin gene clusters encoding for BoNT, NTNHA, p47, BotR and the OrfX1, OrfX2 and OrfX3 proteins were compared between strain Chemnitz (BoNT/A8) and the other BoNT/A subtypes (A1 to A7) and percent identities are given either on nucleic acid level (A) or on amino acid level (B). For comparison the identities to BoNT/E and BoNT/F also located within an *ha* ^-^ *orfX* ^+^ neurotoxin gene cluster are displayed, too. Highest identities (±0.5) are depicted in **bold** and lowest identities (±0.5) are depicted in *italic*. Empty cells indicate that no sequence data exist while n/a denotes the absence of certain genes from the neurotoxin gene cluster.

### Protein sequencing of BoNT/A8

In addition to genomic characterization, the protein sequence of BoNT/A8 was analyzed by high resolution LC-MS/MS. After immunoaffinity enrichment the BoNT was digested with three endopeptidases (AspN, trypsin and chymotrypsin), leading to a sequence coverage of ~67%. MS/MS spectra of the doubly charged precursor ion m/z 923.01 revealed the amino acid sequence ^872^NITNTSILSIVVDKDGR^888^ and verified the arginine insertion at position 888 (Fig. 2B), which led to an additional trypsin cleavage site. Additionally, 57 out of 87 amino acid mutations deduced from nucleotide sequence could be confirmed by this protein sequencing approach (S2 Fig.).

Furthermore, the MS/MS data of the novel subtype were used to challenge the amino acid substitution database recently described [13]. The amino acid substitution database identified nine of the 12 unique point mutations in BoNT/A8 compared to the sequence of BoNT/A1 and the other known subtypes (S1 and S2 Figs.). On this basis the substitution database classified BoNT/A8 as “novel” subtype.

### Recognition by antibodies

**Fig 4:**
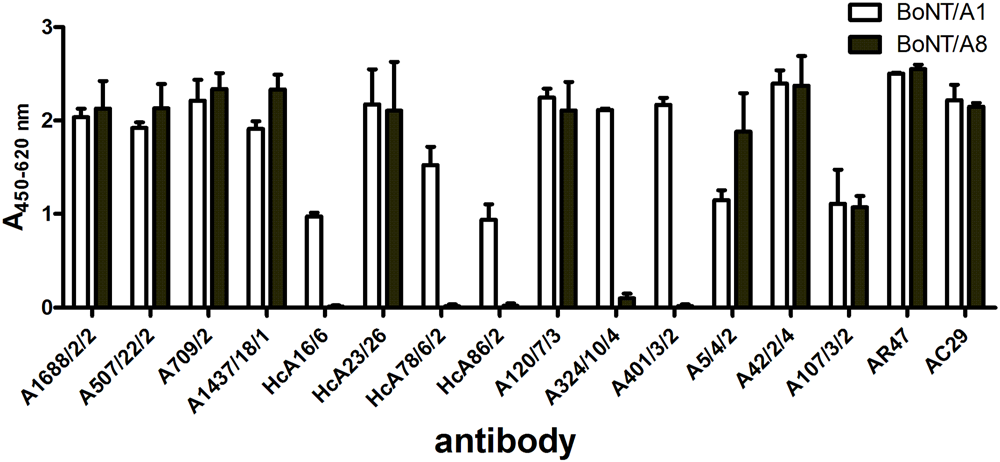
Differential binding of antibodies to BoNT/A1 and BoNT/A8. Native or recombinantly expressed single chain BoNT/A1 and single chain BoNT/A8 were coated onto microtiter plates and tested in an indirect ELISA for capturing a panel of monoclonal and polyclonal antibodies generated in-house. All antibodies shown are monoclonal antibodies except for AR47 and AC29 which are polyclonal antibodies derived from rabbit and chicken, respectively. Shown is the mean (± SD) of three independent experiments performed in duplicates.

We tested whether the observed differences on protein level resulted in an altered antibody recognition pattern: both BoNT/A8 and BoNT/A1 were recombinantly expressed as single-chain molecules, immobilized on a 96-well plate and tested for binding a panel of monoclonal and polyclonal antibodies generated in-house ([47–49] and unpublished data). As shown in Fig. 4, a number of monoclonal antibodies (mAbs) showed no difference in recognizing BoNT/A1 and A8, while others showed a reduced binding or even a complete loss of binding to BoNT/A8 (mAb HcA16/6, HcA78/6, HcA86/2, A324/10, and A401/3).

The antibodies showing a differential binding between BoNT/A1 and BoNT/A8 recognize different epitopes on the BoNT molecule, with mAb HcA16/6, HcA78/6 and HcA86/2 being directed against the H_C_-domain, mAb A401/3 recognizing the LC domain and mAb A324/10 recognizing a yet to be determined conformational epitope on the molecule. Binding to two polyclonal antibodies raised in rabbit (AR47) or chicken (AC29) was not affected (Fig. 4).

### Receptor binding of BoNT/A8

**Fig 5:**
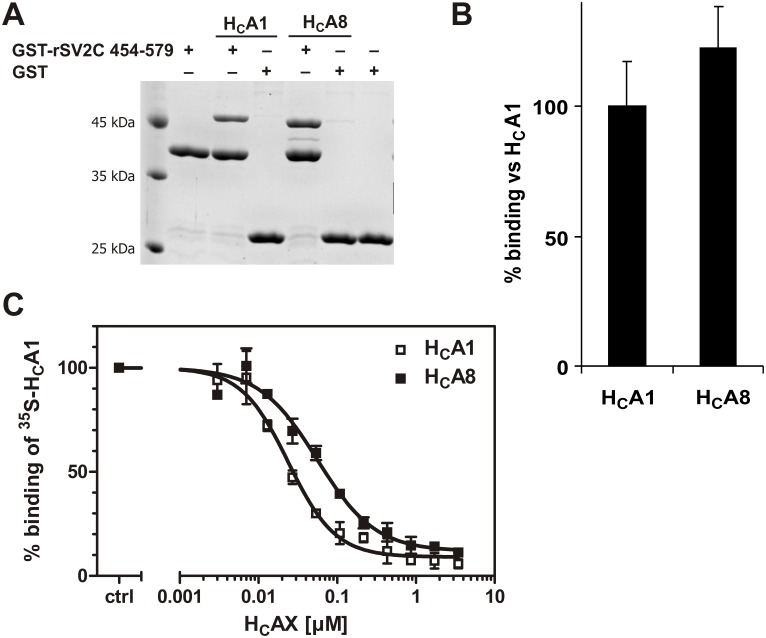
Binding characteristics of BoNT/A8 cell-binding domain H_C_. (A) Binding affinity of H_C_A8 to its synaptic protein receptor rat SV2C in a GST pull-down assay is similar to H_C_A1. 12.5% SDS-PAGE and Coomassie staining of a representative pull-down experiment. (B) Densitometric quantification of bound H_C_-fragments from (A) (n = 4 ± SD). (C) Competition assay: Binding of ^35^S-H_C_A1 to synaptosomes at 4°C in the presence of increasing amounts of recombinant H_C_A1 or H_C_A8, respectively. Bound ^35^S-H_C_A1 was detected by SDS-PAGE and autoradiography (n = 3 ± SD; H_C_A1: EC_50_ = 24 nM (95% CI 20–29 nM), H_C_A8: EC_50_ = 56 nM, 95% CI 43–72 nM)).

Little is known about the functional consequences of the sequence differences of BoNT subtypes. Most of our knowledge regarding binding of BoNT to the synaptic membrane, transcytosis and proteolytic action towards SNAP-25 is based on the archetype BoNT/A1. In our first set of experiments we investigated if differences in the amino acid sequence affect binding to the synaptic cell surface. Binding of BoNT/A follows a two-receptor model in which BoNT/A shows a low affinity interaction towards gangliosides and a high affinity binding to synaptic vesicle glycoprotein 2 (SV2) family members [23]. BoNT/A1 displays the highest affinity to the luminal domain 4 of SV2 isoform C [27]. First we analyzed binding of the recombinant isolated cell binding domain H_C_ to its immobilized protein receptor rat SV2C. The corresponding 126 aa peptide 454–579 has been fused to glutathione-S-transferase (GST) to allow immobilization on GT-matrix [27]. GST-rSV2C 454–579 could equally well precipitate H_C_A1 and H_C_A8 from the supernatant, indicating no change in affinity towards their protein receptor (Figs. 5A and 5B). We then measured the interaction with gangliosides by the interaction of the H_C_-domain with synaptosomes at 4°C. In contrast to an equal interaction with the protein receptor, the binding of radiolabeled ^35^S-H_C_A1 to synaptosomes was inhibited by lower concentrations of recombinant H_C_A1 than those of H_C_A8. Whereas recombinant H_C_A1 displayed an EC_50_ of 24 nM (95% CI 20–29 nM), 2.5-fold more H_C_A8 was required to displace ^35^S-H_C_A1 (EC_50_ = 56 nM, 95% CI 43–72 nM), indicating that H_C_A8 has a lower binding affinity to gangliosides incorporated in neuronal membranes compared to H_C_A1 (Fig. 5C).

### Enzymatic activity of BoNT/A8

**Fig 6:**
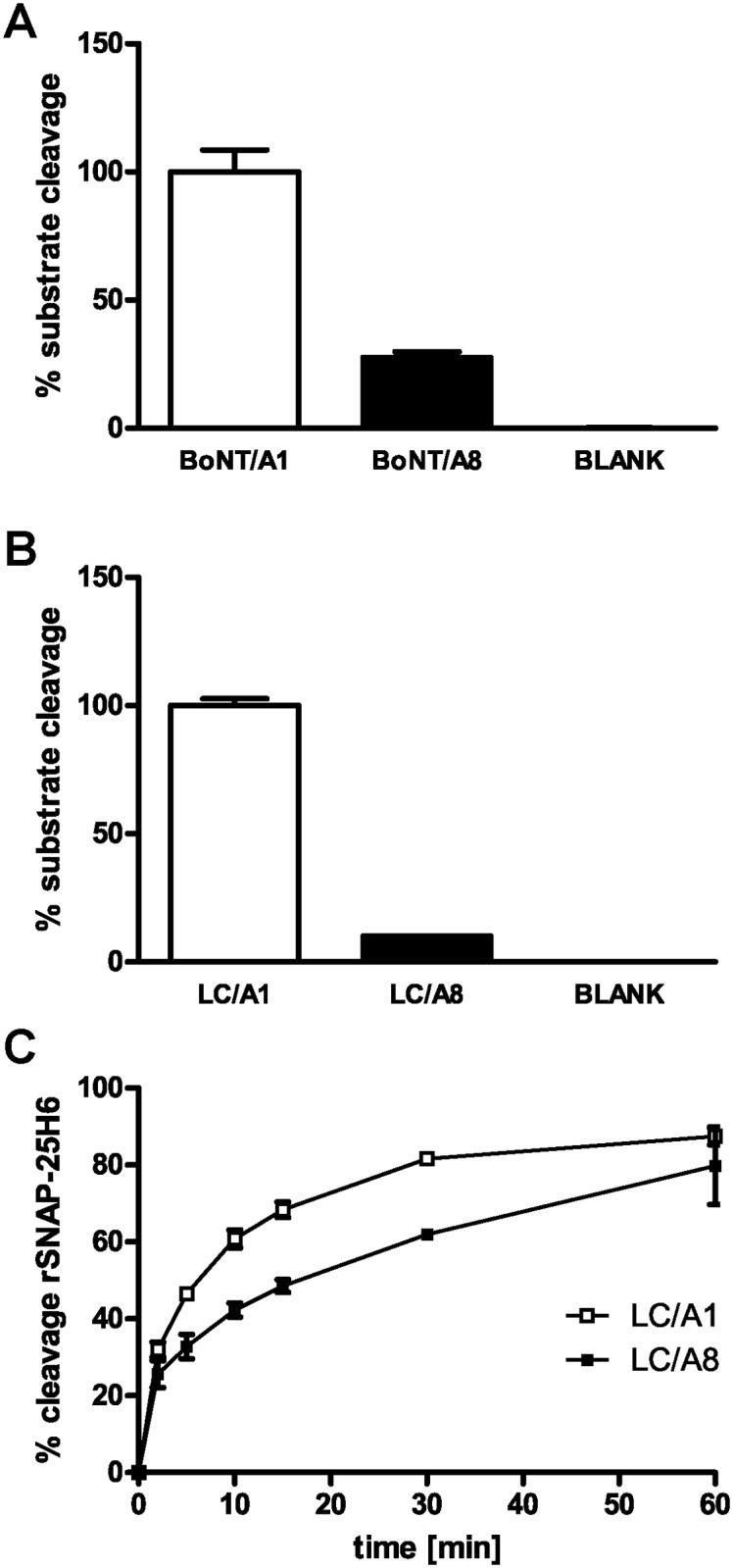
Comparison of the catalytic activity of BoNT/A1 and BoNT/A8. (A) Quantitative mass spectrometric endopeptidase assay of native full-length BoNT/A1 and BoNT/A8 immunoenriched from cell culture supernatants tested for cleavage of a SNAP-25 peptide substrate. (B) Quantitative mass spectrometric endopeptidase assay of recombinant light chains of BoNT/A1 and BoNT/A8 tested for cleavage of human H6SNAP-25 protein. Both diagrams show the percentage of BoNT/A8 substrate cleavage compared to BoNT/A1 (n = 3 ± SD). (C) Time-dependent cleavage of rat SNAP-25H6 (3 μM) by LC/A1 and LC/A8 (0.5 nM). The amount of cleavage product was quantified densitometrically from SDS-PAGE stained by Coomassie (n = 3 ± SD).

After characterization of the binding domain we explored the LC for differences in its catalytic activity. In order to analyze the endopeptidase activity of BoNT/A8 in comparison to BoNT/A1, a combination of immunoaffinity enrichment plus mass spectrometric quantification of substrate cleavage was performed [50]. The native toxins were enriched from cell-free culture supernatants using mAb 4E17.1 coupled to magnetic beads which recognizes the strictly conserved epitope YNQYTEEEK (aa 751–759; [51] and S1 Fig.). Captured toxins were then incubated in an Endopep-MS assay with a cleavable 22-mer SNAP-25 peptide. We found that BoNT/A8 showed only about 28% activity compared to that of BoNT/A1 (Fig. 6A). It has been described that the antibody used for immunocapturing the BoNT might affect the catalytic activity of LC in a subtype-dependent manner [38,39]. To exclude any deleterious effects of the antibody used, we omitted the immunocapture step and compared the activity of recombinantly expressed LC only. LC of BoNT/A1 and BoNT/A8 were incubated with the full-length human SNAP-25 protein, and catalytic activity was determined in an Endopep-MS assay. Consistent with the results obtained with the native full-length molecule we found a dramatic reduction in catalytic activity to about 10% when comparing the activity of LC/A8 to that of LC/A1 (Fig. 6B).

Moreover, the findings of the Endopep-MS assay could be confirmed in an independent experimental setting measuring rat SNAP-25 wild-type cleavage in a time-dependent endopeptidase assay, where the cleavage activity was determined by SDS-PAGE and densitometric quantification of the cleavage product. Again, results clearly corroborated the fact that LC/A8 displays a lower rate of catalysis than LC/A1 (Fig. 6C).

### Biological activity of BoNT/A8

**Fig 7:**
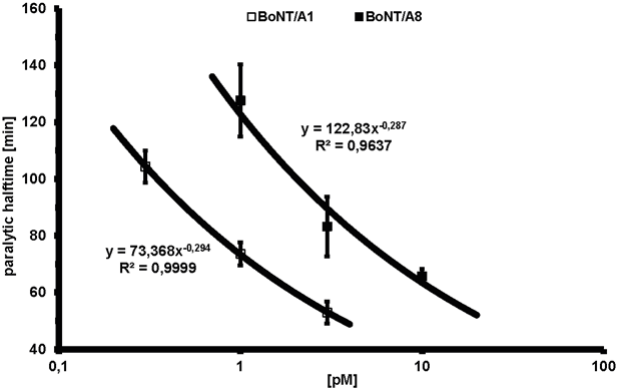
Biological activity of BoNT/A1 and A8. The biological activity of recombinant full-length active BoNT/A1 and BoNT/A8 was determined employing the mouse phrenic nerve hemidiaphragm (MPN) assay. To a three-point dose—response curve (n ≥ 3 ± SD) of each subtype a power function was fitted. To yield an identical paralytic halftime, six-fold higher concentrations of BoNT/A8 are required.

To verify whether the altered characteristics in binding and catalytic activity results in a modified biological activity, the potency of full-length BoNT/A1 and BoNT/A8 molecules—recombinantly expressed as single-chain molecules and activated *in vitro*—was determined employing the MPN assay which represents the physiological site of action of BoNT. Comparison of the three-point dose—response curves (Fig. 7) yielded an about six-fold reduced biological activity of BoNT/A8 versus BoNT/A1, caused by the reduced binding and catalytic properties of BoNT/A8.

## Discussion

Here we describe the isolation and characterization of a novel *C*. *botulinum* strain from a food-borne botulism case near Chemnitz, Germany. Comparison of its BoNT/A sequence to the previously published sequences reveals that it constitutes a novel subtype, designated as the next consecutive number BoNT/A8.

The comparison of the BoNT/A sequences of strain CDC41370 [9] and strain 2008–128 [10] showed at least 2.6% difference on amino acid level to the previously described subtypes A1 to A5 [7,52,53]. Although the differences for both sequences were noted by the authors, no novel subtypes were assigned in their original publications [9,10]. For the sake of consistency we decided to designate these two BoNT/A sequences as subtypes BoNT/A6 (CDC41370) and BoNT/A7 (2008–128), respectively, in this work. BoNT/A8 displays the highest homology (~93.3%) to BoNT/A1, A2 and A5. Interestingly, BoNT/A2 is encoded in an *ha* ^-^ *orfX* ^+^ gene cluster like BoNT/A8 which yields only the 300 kDa M-progenitor toxin complex (M-PTC) comprising BoNT/A and the non-toxic non-haemagglutinin (NTNHA) [54]. Recently, it has been demonstrated that only the fully functional 760 kDa large PTC (L-PTC) comprising the additional dodecameric haemagglutinin (HA) complex displays high oral toxicity [55]. Nevertheless, strain Chemnitz producing BoNT/A8 caused a serious case of food-borne botulism which poses the question whether the three OrfX proteins or other unknown clostridial proteins can adopt the function of the HA complex to achieve high oral toxicity.

Comparison of the protein sequences of BoNT/A8 with BoNT/A1 showed that BoNT/A8 includes an insertion of a single Arg at position 888, rendering A8 with 1297 aa the longest of all known BoNT/A subtypes. Noteworthy is also the change of the final amino acid from leucine to glutamine which could only be identified by 454 shotgun data analysis; initially, the primer BoNT A-6R described by Hill *et al*. [7] changed the terminal DNA triplet to a codon encoding leucine, as present in A1 to A7.

Interestingly, one other published *C*. *botulinum* strain, CDC2171 of subtype A2 (GenBank: GQ241939) [45], also consists of 1297 aa and comprises a glutamic acid residue inserted at position 888 and glutamine at position 1297. Noteworthy, this particular strain showed the highest overall identity of 94.3% towards strain Chemnitz. A detailed comparison on the level of subdomains of CDC2171 with strain Chemnitz revealed that in particular the H_CN_ and H_CC_ domains were highly identical (95.1% and 98.0% identity, respectively) while the H_N_ domain showed a lower identity of 89.6% and the identity of the LC (96.8%) was close to subtypes BoNT/A1, A5, A6, and A7.

While the BoNT/A8 itself showed highest identity with BoNT/A subtypes, we intriguingly found the highest identity of the other genes in the neurotoxin gene cluster, namely *ntnha*, *orfX1–3*, *BotR* and *p47*, with that of strain Langeland encoding BoNT/F1. However, the individual subdomains of BoNT/A8 are not highly related to BoNT/F1. This finding allows the interpretation that BoNT/A8 is localized in a BoNT/F1 gene cluster.

It has been recognized before that BoNT/A and F producing bivalent strains exist [12,56], even a trivalent BoNT/A2, F4 and F5 producing strain has been identified [57–59]. Notably, recent analysis of the proposed novel serotype H indicated that this BoNT could also be described as F5A1 mosaic form [60,61]. Indeed, when we compared the H_CC_ domain of BoNT/A8 with the one of the proposed BoNT/H (GenBank: KGO15617) we found 95.1% identity on amino acid level whereas the identities for the other domains were significantly lower (LC: 31.2%, H_N_: 39.0%, H_CN_: 67.1%). On the basis of these findings one could speculate that recombination events or interchange of *bont* or other toxin cluster genes occurs more likely between serotypes A and F and that the neurotoxin gene cluster of strain Chemnitz might be the result of a transfer of *bont/a8* into the *bont/f1* gene cluster. This can only be corroborated by a detailed comparison of the backbone of strain Chemnitz versus strain Langeland in the future.

It is an ongoing debate among the BoNT community what differences qualify a toxin gene to be called a novel, distinguishable subtype. Different definitions have been proposed, e.g. differential recognition and neutralization by antibodies, different primary sequence—with the inherent question of where to set the threshold between different subtypes—or different functional aspects of individual toxin variants/subtypes. The current definition of subtypes based on differences on amino acid level of at least 2.6% [6] is to a certain degree arbitrary. Also, depending on the serotype, different thresholds have been set; e.g. the subtypes E1 to E3 differ only between 1 to 3% [7]. Historically, the typing of BoNTs is nearly as old as its discovery. Soon after van Ermengen had isolated and described the first strain [62], it was noted that the toxic supernatant of this strain differed from that of another strain isolated from bean salad in Darmstadt [63] not only biochemically but also antigenically, as the corresponding antitoxins did not cross-neutralize each other [64]. Thus the first two serotypes were born. However, the very recent issue of subtyping of serotypes is more difficult, since we and others noted a large variability among the sequences of specific BoNT serotypes, including amino acid variations within a given subtype [6,7,13]. Generally, a few point mutations at critical positions might interfere with recognition of antibodies or modify functional characteristics, even if the distance to other subtypes is less than 2.6%. So in one extreme, if each different sequence would be designated as individual subtype, we might end up with countless BoNT subtypes. On the other hand, no subtyping would lead to a confusing situation and would severely impede comparison of different study results.

There is no question that differences in the sequence might significantly affect detection either on DNA or protein level, and in fact this has been recognized for some subtypes in the past [6,36,38,65] and can also be demonstrated for BoNT/A8 versus BoNT/A1 with a panel of different antibodies. However, one of the most intriguing questions is whether the observed sequence variation in BoNT/A8 does affect the biological activity. The overall functional activity of BoNT comprises the catalytic activity of the LC domain, the cell-binding activity of the H_C_ domain and the translocation activity of the H_N_ domain. Some predictions can be made based on the amino acids identified to be essential for the process (e.g. catalysis, substrate or receptor binding). The limitation of such a theoretical approach lies in the unpredictability of the complex three-dimensional interactions within the protein structure and a lack of knowledge of all atomic interactions during pathogenesis. So far, there is little experimental data on functional activity of known subtypes. Henkel *et al*. compared the catalytic activity of the isolated LC from BoNT/A1, A2, A3 and A4. While LC/A1 and LC/A2 were found to have similar activity towards GST-SNAP-25 (aa 141–206), LC/A3 was 2-fold and LC/A4 23-fold less active [40]. However, others found substantial time-dependent differences in the cleavage efficacies between subtypes BoNT/A1 to A5. [66]. Additionally, Wang *et al*. identified different cleavage kinetics for BoNT/A1 and A5 [41].

When we compared the catalytic activity of either the native full-length BoNT/A8 from culture supernatants or the isolated recombinant LC from BoNT/A8 with the respective molecule of BoNT/A1, we found its activity towards full-length hSNAP-25 or a SNAP-25-derived peptide significantly reduced to about 10–28% of the activity of BoNT/A1 in Endopep-MS assays (Fig. 6A and B). In a different approach with full-length rat SNAP-25, a different buffer system and a different read-out the LC/A8 activity also displayed reduced activity compared to LC/A1 but only by 10–20% (Fig. 6C). While the rat and human SNAP-25 amino acid sequences are identical, the buffer system and assay time have been found to influence the LC activity greatly [66–68]. Indeed, for the Endopep-MS assay (Fig. 6A and B) a buffer consisting of 20 mM HEPES pH 7.3, 10mM dithiothreitol, 20 μM ZnCl_2_ and 1 mg/mL BSA [50] was used, whereas 150 mM potassium glutamate and 20 mM HEPES-KOH, pH 7.2 [69] was used for the time-dependent endopeptidase assay (Fig. 6C). In fact, dithiothreitol, ZnCl_2_ and BSA have been shown to have a major impact on the catalytic activity [67,68]. So the differences observed in Fig. 6A/B versus Fig. 6C with respect to catalytic activity might well reflect the different reaction conditions used to compare BoNT/A8 and BoNT/A1. Importantly, irrespective of the individual assay conditions used to compare both molecules in different endopeptidase assay formats, we always observed a reduced activity of BoNT/A8, underlining the validity of the results in different *in vitro* functional assays. In BoNT/A8, 12 amino acid residues within the 438 amino acids of LC are substituted compared to BoNT/A1. Only four of those residues (T13, I17, K55 and N270) also differ from LC/A2 (S1 Fig.) which is nearly as catalytically active as LC/A1 [40]. Superimposition of a structural model based on the amino acid sequence of LC/A8 1–438 on the co-crystal structure of LC/A1 and SNAP-25 [70] as well as a LC/A2 as monomer [71] revealed only few substituted residues in the vicinity of the substrate-binding cleft. Interestingly, Arg191 of SNAP-25 which follows just after the SNAP-25 loop 187–189 and is in close proximity to the scissile peptide bond (Gln197-Arg198) is coordinated by E171 in LC/A1. In LC/A8 an aspartate is found at position 171, which could lead to an impaired salt bridge formation; however, LC/A2 also comprises D171. Furthermore, on the opposite rim of E171 the residue E55 is exclusively replaced by a lysine in LC/A8. Such a charge reversal was expected to repel Arg191 of SNAP-25 and thereby weaken the substrate binding of SNAP-25 to LC/A. However, substituting E55 in LC/A1 for lysine did not impair time-dependent substrate cleavage of SNAP-25 (aa 1–206). Accordingly, the mutant LC/A8 K55E neither showed an increased cleavage rate compared to LC/A8 wild type nor reached the activity of LC/A1 wild type (Bachelor thesis Alexander Bollenbach [72]).

While the catalytic activity is one major factor of the toxin’s biological toxicity, receptor binding contributes as well and was therefore analyzed in detail for BoNT/A8 versus BoNT/A1. For high affinity binding to the synaptosomal cell membrane two different binding events, namely binding to a ganglioside and the synaptic vesicle glycoprotein 2 (SV2), have been identified [23]. It has been shown that isoform SV2C is most crucial for uptake of the BoNT molecule [27]. The binding of the H_C_-fragments of BoNT/A1 and A8 to SV2C was comparable in an *in vitro* pull down assay (Fig. 5). This is in accordance with amino acids identified in the co-crystal structure being critical for the interaction of BoNT/A1 with human SV2C, namely T1145, T1146 and Y1149 [73], which are conserved between BoNT/A1 and BoNT/A8 (T1146, T1147 and Y1150 due to R888 insertion). However, R1156 thought to interact with F563 of human SV2C is found exclusively in BoNT/A1 and only BoNT/A2 comprises E1156 whereas all other known subtypes including BoNT/A8 display a methionine at position 1156/7 which still permits interaction with hydrophobic residues such as F563. R1294/5 contributing to the positive surface charge [73] is converted to serine in BoNT/A2, A3, A5, A7, and A8 but to lysine in BoNT/A6. Thus, none of the other BoNT/A subtypes contains R1156 and R1294. We found that in accordance with this loss of surface charge in the SV2 binding site, BoNT/A8 showed reduced salt-dependency in the interaction with rat SV2C as measured by surface plasmon resonance (Daniel Stern, Robert Koch-Institut). In a more recent publication Strotmeier and colleagues identified further residues important for the interaction with SV2C [74]. Three of the identified positions whose mutation alters SV2C binding of BoNT/A1 (N905Y, T1063P, H1064G) divert in BoNT/A8 (N906S, T1064P, H1065R). The exchange N906S introduces a less bulky side chain than the tested N905Y and as such may be of little importance. The homologous substitution of the basic histidine at 1065 by the basic arginine might also not influence SV2C binding. However, T1063P greatly reduces binding of BoNT/A1 to rat SV2C in the pull-down assay, but no such reduction was seen for BoNT/A8 in a very similar experimental setting, which might be due to a compensation by other residue exchanges.

While we did not observe differences in SV2C binding experimentally, we found the interaction of H_C_A8 with gangliosides in neuronal membranes markedly reduced. The single ganglioside-binding site (GBS) of BoNT/A is formed by 12 residues and contains the conserved GBS motif E…H…SXWY…G [23]. All six residues are identical in BoNT/A1 and BoNT/A8. Only L1278, a less important contact point, is replaced by F1279 in BoNT/A8 which introduces an aromatic functionality but still maintains the hydrophobic character of this part of the ganglioside-binding pocket. However, the binding to synaptosomes at 4°C which is predominantly mediated via interaction with gangliosides is clearly reduced for BoNT/A8, which cannot be explained by the L1279F exchange. In the neighborhood of the GBS the surface-exposed loop 1271–1274 (IERS) is mutated to V^1272^GKA^1275^ in BoNT/A8. Due to the short carbohydrate chain of gangliosides, the GBS-surrounding surface will be in close proximity of the membrane surface, and a negative influence of the altered loop 1272–1275 seems reasonable. Furthermore, whereas in the H_CC_-domain 15 of the 205 residues are substituted in BoNT/A8, the H_CN_ even displays 38 exchanges including the insertion of R888. A role of the H_CN_ domain in phospholipid binding has been suggested, although no individual residues could be identified [75]. Hence it is possible that the larger differences in the H_CN_ domain also contribute to the clearly decreased binding of H_C_A8 to neuronal membranes. In the above-mentioned recent study of Strotmeier *et al*. [74] some residues of importance for BoNT/A1 toxicity are described. E.g. Q915R showed little effect on SV2C binding but a 50% reduced toxicity. In BoNT/A8 the Q915 is replaced by lysine, which also introduces a positive charge. Although speculative, this exchange might compromise the interaction with the cell surface or simply hinder certain conformational changes occurring during binding and/or translocation.

When comparing two proteins differing by one amino acid only, a potential assignment in functional consequences is straightforward. In our situation, however, where a number of residues differ simultaneously, interpretation is rather difficult. Individual effects caused by one amino acid exchange might be reversed by another one; also previously unrecognized residues might have an important impact on protein—protein interaction when replaced by another amino acid. Thus, deduction of the functional activity of a protein based on amino acid sequence alone can be misleading and needs experimental investigation.

Due to the complex interplay of cell binding, uptake, transcytosis and catalytic activity, predictions on the overall toxicity based on the activity of single domains can be inaccurate. Whereas Henkel *et al*. found the catalytic activities of the LC from A1 and A2 comparable, others described more profound differences of the full-length molecules in cell culture [66,76,77] or *in vivo* [78,79]. To test whether the observed differences in catalytic activity and ganglioside binding affects the overall toxicity of BoNT/A8, both the novel subtype and BoNT/A1 were expressed as full-length proteins in *E*. *coli* and tested in an MPN assay which measures the neurotoxicity in an *ex vivo* approach. Indeed, BoNT/A8 turned out to have a profound reduction in biological activity (about 6-fold) as compared to BoNT/A1, thus extending the differences observed between single domains onto the full-length molecule.

In summary, we describe and characterize the novel subtype BoNT/A8 that was associated with a case of food-borne botulism in Germany. To our knowledge, this is the first comprehensive description of a novel BoNT subtype providing information both on the genomic organization of the neurotoxin gene cluster and information on the functional characteristics of the toxin’s domains based on different technical approaches. Finally, even though the toxin produced a severe case of food-borne botulism in the patient, the determination of the overall toxicity of the novel subtype demonstrated a significant reduction in neurotoxicity as compared to BoNT/A1. Independent of the definition criteria of a novel subtype, our BoNT/A8 has to be designated as new subtype since it can be distinguished from other BoNT/A subtypes based on (i) sequence variation larger than 2.6%, (ii) different antibody recognition pattern as well as (iii) altered functional/biological activity.

Our results show that subtyping of BoNT is highly relevant and necessary to effectively fight the very sophisticated BoNT molecule. While a reduction in biological activity as identified in this novel subtype does not cause concern under the aspect of biosecurity, the opposite might well be found in a yet unknown subtype. On the other hand, identification and in-depth characterization of BoNT/A subtypes might pave the way for developing novel therapeutics for different indications and medical applications and will also support the development of appropriate BoNT countermeasures.

## Materials and Methods

### Ethics statement

This study does not comprise animal experiments according to the German Protection of Animals Act (TierSchG dated 13.07.2013) and European Union Directive 2010/63/EU for the protection of animals used for experimental purposes. NMRI mice for the MPN assay were obtained from Janvier SAS (St. Berthevin Cedex, France). According to §4.3 (killing of animals for scientific purposes, TierSchG) animals were anesthetized with carbon dioxide and sacrificed by trained personnel before dissection of organs. All efforts were made to minimize suffering. This project no. §4.3/19 was approved by the animal welfare officer of the Central Animal Laboratory of the Medizinische Hochschule Hannover. Consumption of animals was reported yearly to the animal welfare officer and to the local authority, Veterinäramt Hannover, Germany.

### Bacterial strains

Strains used in the present study were NCTC 7272 (ATCC 19397) expressing BoNT/A1 and strain Chemnitz expressing BoNT/A8 (own isolate, Robert Koch-Institut; [44]). Clostridia strains were cultured in tryptone-peptone-glucose-yeast extract (TPGY) broth for up to 4 days at 32°C in an anaerobic workstation (MACS 500, Don Whitley Scientific, West Yorkshire, United Kingdom). Cell-free supernatants were obtained from clostridial cultures after centrifugation (12,000 × *g*, 5 min, 4°C) and filtration through a 0.2 μm filter.

For sequence comparison the following Genbank entries were used throughout this work: Subtype A1, strain “Hall 174” (ATCC 3502) GenID NC_009495.1 [80,81]; subtype A1(B), “Hall 2447” (NCTC 2916) X52066, AY497357, NZ_ABDO02000001 [81–83]; subtype A2, “Kyoto-F”, NC_012563.1 [84]; subtype A3, “Loch Maree”, NC_010520.1 [85,86]; subtype A4, “657Ba”, NC_012658 [86]; subtype A5, “H04402 065”, NC_017299 [87,88]; subtype A6, “CDC41370”, FJ981696 [9]; subtype A7, “2008–148”, JQ954969 [10]; subtype E3, “Alaska E43”, NC_010723 [89]; and subtype F1, “Langeland”, NC_009699 [90].

### DNA isolation and sequencing

DNA from TPGY cultures was extracted with the DNeasy Blood and Tissue Kit (Qiagen, Hilden, Germany) according to the manufacturer’s protocol for Gram-positive bacteria.

Sanger Sequencing of 16S *rDNA* and *bont/a* genes was performed as described [7]. For BoNT/A8 an alternative A-1F primer sequence CGGTAAATATATATGTTTATCTAT was used.

For whole genome sequencing the 454 FLX Titanium kit (Roche, Branford, CT) was used on a GS FLX+ System (Roche) after quality assessment of the extracted DNA with the Quant-iT PicoGreen dsDNA Assay Kit (Invitrogen, Darmstadt, Germany). Sequence data of the whole BoNT/A8 gene cluster is deposited at GenBank under accession no. KM233166. Please note that recently a sequence encoding a highly similar BoNT/A has been deposited independently under GenBank accession number KF667385.

### Sequence analysis

Genome sequences were assembled using MIRA [91]. The contigs of the genome assembly as well as Sanger sequences were loaded into the Geneious software package (Biomatters, Auckland, New Zealand) and a contig harboring the neurotoxin gene cluster was identified. The core neurotoxin cluster was elongated by assembling genome sequencing reads over several rounds until the insertion sites into the chromosome were identified.

Homologous gene sequences were compared to the NCBI database entries using the BLAST algorithm, and pairwise identities of the aligned sequences were calculated with Geneious applying the MAFFT algorithm [92].

### Toxin extraction

For BoNT/A extraction from culture supernatants of strain Chemnitz monoclonal antibody (mAb) 4E17.1 was used from the laboratory of James Marks at the University of California, San Francisco, CA, USA [50,51]. The mAb was immobilized on Protein G Dynabeads as described earlier [13]. 20 μL of antibody-coated beads were mixed with a solution of 500 μL of BoNT/A culture supernatant and 50 μL of 10× phosphate buffered saline (PBS) with 0.01% Tween (PBS-T) buffer. The mix was incubated for 1 h at room temperature under constant agitation and washed twice with 1 mL of HEPES buffered saline with EDTA and Tween 20 (HBS-EP, Teknova, Hollister, CA) and once in 100 μL of water.

### Antibodies and indirect ELISA

For binding of recombinantly expressed single chain BoNT/A1 and BoNT/A8 against a panel of in-house generated monoclonal and polyclonal antibodies ([47–49] and unpublished data), the following antibodies were used: mAb A1688/2, mAb A507/22 and mAb A1437/18 generated by immunization with BoNT/A1 toxoid and native BoNT/A1 [48]; mAb A709/2 generated by peptide immunization (aa 72–95; YYDSTYLSTDNEKDNYLKGVTKLF); mAb HcA16/6, mAb HcA23/26, mAb HcA78/6 and mAb HcA86/2 generated by immunization with recombinant H_C_A1 fragment [49]; mAb A120/7, mAb A324/10 and mAb A401/3 generated by DNA immunization [93] followed by boost with BoNT/A1 toxoid; mAb A5/4, mAb A42/2 and mAb A107/3 generated by immunization with recombinant H_C_A1 fragment and boost with BoNT/A1 toxoid. Polyclonal antibodies AR47, AC29 have been described before [47,48].

Native BoNT/A1 (Metabiologics Inc. Madison, WI) or recombinant single chain BoNT/A1 or A8 (500 ng/mL) were coated in PBS containing 3 μg/ml of BSA overnight onto 96-well microtiter plates (Nunc MaxiSorp, Thermo Scientific, Germany). Unspecific binding was blocked with casein buffer (Senova, Weimar, Germany) for 1 h at room temperature. After washing, toxins were incubated for 2 h at room temperature with anti-BoNT/A1-specific monoclonal and polyclonal antibodies at 10 μg/mL in blocking buffer. The ELISA was developed by incubation (1 h) with biotin-labeled anti-species secondary antibodies (Dianova, Hamburg, Germany) diluted in blocking buffer and detection with Streptavidin-PolyHRP40 conjugate (0.5 ng/ml, Senova) and tetramethylbenzidine (SeramunBlau slow, Seramun, Heidesee, Germany).

### Protein sequencing

Toxin was extracted from culture supernatants as above. Beads were reconstituted in 15 μL of 0.5× Tris-PBS buffer (0.5× PBS, 10 mM tris(hydroxymethyl)aminomethane (Tris), pH 7.4), and toxins on the beads were digested with 1 μL of 0.1 μg/μL AspN enzyme for 5 min at 52°C. Following digestion, the supernatant was removed from the beads and supplemented with 2 μL of 10% trifluoroacetic acid (TFA). The beads were reconstituted in 15 μL of 50 mM ammonium bicarbonate, pH 7.5 (tryptic buffer), and 2 μL of 0.5 μg/μL trypsin and digested for 5 min at 52°C. Again, the supernatant was removed from the beads and supplemented with 2 μL of 10% TFA. Finally, the beads were reconstituted again in 15 μL of tryptic buffer and 1 μL of 0.2 μg/μL chymotrypsin and digested for 5 min at 52°C. The supernatant was removed and supplemented with 2 μL of 10% TFA.

Chromatographic separation of peptides and mass spectrometric analysis by Fourier transform ion cyclotron resonance (FT-ICR)-MS as well as database searches were performed as described earlier [13], with the exception that an in-house amino acid substitution database for BoNT/A1 was used.

### Peptides and proteins

Wild-type human SNAP-25 aa 1–206 *N*-terminally fused to His6tag (hH6SNAP-25) was obtained from ATGen, Sampyeong-dong, South Korea. Recombinant rat SNAP-25 fused to a *C*-terminal His6tag (rSNAP-25H6) was expressed as described previously [94]. Recombinant luminal domain 4 (aa 454–579) of rat SV2C fused to GST (GST-rSV2C 454–579) was expressed and isolated as described previously [27]. The genes encoding aa 1–438 (LC) of BoNT/A1 (GenBank ID AAA23262) and BoNT/A8, aa 827–1297 of BoNT/A8 (H_C_A8) and full-length BoNT/A8 were amplified by PCR and inserted into a pQE-based expression plasmid (Qiagen, Hilden, Germany) encoding a *C*-terminal Streptag yielding pLC-A1S, pLC-A8S, pH_C_A8S and pBoNTA8S. The active full-length single chain (sc) BoNT/A1 and scBoNT/A8 were expressed recombinantly in *E*. *coli* in a biosafety level 2 containment (project identifier GAA Hannover A/Z 40654/3/123). Single-step purification of LC/A1S, LC/A8S, H_C_A1S, H_C_A8S, scBoNTA1S and scBoNTA8S was performed as described previously [25]. scBoNT/A were activated into the dichain BoNT/A by a *C*. *botulinum* culture supernatant extract and subjected to gelfiltration in 50 mM sodiumphosphate, pH 7.4, 150 mM NaCl. The LC/AX were dialyzed against toxin assay buffer (150 mM potassium glutamate and 20 mM HEPES-KOH, pH 7.2). H_C_AX were kept in 150 mM NaCl, 100 mM Tris-HCl, pH 8.0 and scBoNT/AX in 100 mM Tris-HCl, pH 8.0. All proteins were flash frozen in liquid nitrogen, and stored at—70°C.

### GST pull-down assay

Glutathione-S-transferase (GST) or GST-rSV2C 454–579 fusion protein (150 pmol each) immobilized to 10 μL of glutathione-sepharose-4B matrix was incubated with recombinant H_C_-fragment of BoNT/A1 and A8 (100 pmol each), respectively, in a total volume of 200 μL of 20 mM Tris—HCl, 80 mM NaCl, pH 7.4, supplemented with 0.5% Triton X-100 (Tris/NaCl/Triton) buffer for 2 h at 4°C. Beads were collected by centrifugation and washed twice with 200 μL of Tris/NaCl/Triton buffer. Beads were incubated for 20 min at 37°C in SDS sample buffer, analyzed by 12.5% SDS-polyacrylamide gel electrophoresis (SDS-PAGE), detected by Coomassie blue staining, densitometrically quantified as binding in mol% of GST-rSV2C and set in ratio to H_C_A1 binding.

### Binding of H_C_-fragments to synaptosomes

Rat brain synaptosomes were freshly prepared and H_C_-fragment of BoNT/A1 (^35^S-H_C_A1) was radiolabeled by *in vitro* transcription/translation as previously described [25]. ^35^S-H_C_A1 was bound in the presence of increasing concentrations of recombinant H_C_A1S and H_C_A8S (0.003–3.5 μM) to synaptosomes in a total volume of 100 μL of physiological buffer (140 mM NaCl, 5 mM KCl, 1 mM MgCl_2_, 1 mM CaCl_2_, 20 mM HEPES, 10 mM glucose, 0.5% BSA, pH 7.4) with the final synaptosomal protein adjusted to a concentration of 10 mg/ml for 2 h at 4°C. Controls were performed with samples lacking synaptosomes and/or recombinant H_C_A. After incubation, synaptosomes were collected by centrifugation (5,000 × *g*; 5 min) and unbound material in the supernatant fraction was discarded. The pellet fractions were washed two times each with 100 μL of physiological buffer and incubated for 20 min at 37°C in SDS sample buffer and subsequently subjected to SDS-PAGE and autoradiography. Bound ^35^S-H_C_A1 was quantified with the Advanced Image Data Analyzer (AIDA 2.11) software (Raytest, Straubenhardt, Germany) and calculated after subtraction of the value obtained for control sample in the absence of synaptosomes as the percentage of the total ^35^S-H_C_A1 in the assay. The inflection points of the binding curves were determined by fitting the data to the sigmoidal dose—response equation with variable slope, bottom constraint >0, top constraint <100 using Prism 4.03 (GraphPad Software, La Jolla, CA, USA) and expressed as EC_50_ values in units of μM.

### Mass spectrometric Endopeptidase activity assay (Endopep-MS)

The Endopep-MS reaction was performed as described previously [50] with a few modifications. After immunoextraction of the toxin from culture supernatants as described the beads were incubated with the 4 nmol peptide substrate (Biotin-KGSNRTRIDQGNQRATRXLGGK-Biotin where X represents norleucine) for 40 min in 40 μL of reaction buffer (20 mM HEPES pH 7.3, 10 mM dithiothreitol, 20 μM ZnCl_2_, 1 mg/mL BSA). 18 μL of the sample was combined with 2 μL of 1% formic acid and 2 μL of a 100 μM internal standard peptide (RA(+7)TRXLGGK-Biotin) for SNAP-25 peptide quantification. LC-MS/MS analysis of the cleavage products was performed as described by Parks *et al*. [95]. Additionally, the antibody-coated beads which were used for BoNT extraction were washed twice with 100 μL of water and reconstituted in 20 μL of tryptic buffer and 2 μL of 0.5 μg/μL trypsin and digested for 5 min at 52°C. After incubation 20 μL of each sample was combined with 2 μL of 1% formic acid and 2 μL of a 1 μM internal standard peptide for BoNT quantification (L(+7)VASNWYNR). LC-MS/MS analysis of the BoNT peptide fragment was performed on an AB Sciex 5500 triple quadrupole mass spectrometer (AB Sciex, Foster City, CA, USA).

For the Endopep-MS with human His-tagged full-length SNAP-25 (hH6SNAP-25) protein 1 nM recombinant light chains LC/A1S and LC/A8S were incubated in 20 μL of reaction buffer (20 mM HEPES (pH 7.3), 10 mM dithiothreitol, 20 μM ZnCl_2_ and 1 mg/mL BSA) with 4 μM hH6SNAP-25 protein. The samples were incubated at 37°C for 30 min and then inactivated at 95°C for 10 min in a PCR cycler. Afterwards 2 μL of 1% formic acid and 2 μL of 100 μM internal standard (RATKML(+7)GSG) were added for SNAP-25 protein quantification. LC-MS/MS analysis was performed on an EASY nanoLC coupled to a linear ion trap (LTQ) Orbitrap Discovery (both Thermo Scientific, Bremen, Germany).

### Time-dependent SNAP-25 endopeptidase assay

In time-dependent SNAP-25 endopeptidase assays, 3 μM rSNAP-25H6 was incubated in the presence of 0.5 nM LC/A in a total volume of 120 μL in toxin assay buffer at 37°C. Aliquots (15 μL) were withdrawn at specified time intervals. Reactions were stopped by mixing with 5 μL of 4-fold Laemmli buffer. Samples were boiled for 2 min and subjected to 15% SDS-PAGE. Proteins were visualized by staining with Coomassie blue and quantified by densitometry.

### Mouse phrenic nerve (MPN) hemidiaphragm assay

The MPN assay was performed as described previously [74]. In short, the phrenic nerve was continuously stimulated at 5–25 mA with a frequency of 1 Hz and with 0.1 ms pulse duration. Isometric contractions were transformed using a force transducer and recorded with VitroDat Online software (FMI GmbH, Seeheim, Germany). The time required to decrease the amplitude to 50% of the starting value (paralytic half-time) was determined. To compare the altered neurotoxicity of BoNT/A8 with BoNT/A1 three point concentration—response curves determined minimum in triplicates were compiled to which the following power functions could be ascribed: y(BoNT/A1S; 0.3, 1, 3 pM) = 73.368x^–0.2941^, R^2^ = 0.9999 and y(BoNT/A8S; 1, 3, 10 pM) = 122.83x^–0.2867^, R^2^ = 0.9637. To yield an identical paralytic half-time of 75 min, 6.0-fold higher concentrations of BoNT/A8 were required.

## Supporting Information

S1 Fig Alignment of amino acid sequences from subtypes BoNT/A1 to BoNT/A8. The figure displays an alignment of amino acid sequences from subtypes BoNT/A1 to BoNT/A8 with light chain indicated in dark grey and heavy chain indicated in light grey; unique amino acid differences in BoNT/A8 based on the representatives of BoNT/A1 to A7 are marked in red; arginine insertion in position 888 is marked in green; amino acids important for catalytic activity are marked in magenta; essential amino acids for ganglioside-binding (E…H…SXWY..G) motif are given in turquoise.
(DOCX)

S2 Fig Amino acid sequence coverage after combined endopeptidase digest and tandem mass spectrometry analysis. Amino acid coverage of BoNT/A8 as observed after combined endopeptidase digest and LC-MS/MS is shown in amber; unique amino acid differences present in BoNT/A8 based on the representatives of BoNT/A1 to A7 are marked in red; mutations at positions also found in other BoNT/A subtypes are marked in yellow (see also Fig. 2); arginine insertion at position 888 is marked in green.
(DOCX)
