# Supplementary material for: Isolation and Functional Characterization of the Novel Clostridium botulinum Neurotoxin A8 Subtype
Source: PLoS One. 2015 Feb 6;10(2):e0116381. doi: 10.1371/journal.pone.0116381 (PMC4320087; doi:10.1371/journal.pone.0116381)
Supplement: S1 Fig — The figure displays an alignment of amino acid sequences from subtypes BoNT/A1 to BoNT/A8 with light chain indicated in dark grey and heavy chain indicated in light grey; unique amino acid differences in BoNT/A8 based on the representatives of BoNT/A1 to A7 are marked in red; arginine insertion in position 888 is marked in green; amino acids important for catalytic activity are marked in magenta; essential amino acids for ganglioside-binding (E…H…SXWY..G) motif are given in turquoise. (DOCX) [file pone.0116381.s001.docx]

**Figure S1:** Alignment of amino acid sequences from subtypes BoNT/A1 to BoNT/A8

10 20 30 40 50 60 70 80 90 100

....|....|....|....|....|....|....|....|....|....|....|....|....|....|....|....|....|....|....|....|

**ATCC_3502_(A1)**  **MPFVNKQFNYKDPVNGVDIAYIKIPNAGQMQPVKAFKIHNKIWVIPERDTFTNPEEGDLNPPPEAKQVPVSYYDSTYLSTDNEKDNYLKGVTKLFERIYS**

**NCTC2916_(A1(B)** **.Q........................V.........................................................................**

**Kyoto_F_(A2)**  **....................................................................................................**

**Loch_Maree_(A3)** **..........R............................EGV.................................................I...D....**

**657Ba_(A4)**  **..........N..E..............K............V.......I......V............I.....A...............I........**

**H04402065_(A5)**  **.L..................................................................................................**

**CDC41370_(A6)**  **....................................................................................................**

**2008-148_(A7)**  **.................................................I..................................................**

**Chemnitz_(A8)**  **............T...I.....................................K.............................................**

110 120 130 140 150 160 170 180 190 200

....|....|....|....|....|....|....|....|....|....|....|....|....|....|....|....|....|....|....|....|

**ATCC_3502_(A1)**  **TDLGRMLLTSIVRGIPFWGGSTIDTELKVIDTNCINVIQPDGSYRSEELNLVIIGPSADIIQFECKSFGHEVLNLTRNGYGSTQYIRFSPDFTFGFEESL**

**NCTC2916_(A1(B)** **....................................................................................................**

**Kyoto_F_(A2)**  **......................................................................D.............................**

**Loch_Maree_(A3)** **.G......SF..K.........................E.G............T................D.F...........................**

**657Ba_(A4)**  **........I...........GK..............I..L.D.........A......N..ESQ.S..RDD......................V......**

**H04402065_(A5)**  **.E....................................................................D.............................**

**CDC41370_(A6)**  **....................................................................................................**

**2008-148_(A7)**  **.............................................................N........D.............................**

**Chemnitz_(A8)**  **......................................................................D.............................**

210 220 230 240 250 260 270 280 290 300

....|....|....|....|....|....|....|....|....|....|....|....|....|....|....|....|....|....|....|....|

**ATCC_3502_(A1)**  **EVDTNPLLGAGKFATDPAVTLAHELIHAGHRLYGIAINPNRVFKVNTNAYYEMSGLEVSFEELRTFGGHDAKFIDSLQENEFRLYYYNKFKDIASTLNKA**

**NCTC2916_(A1(B)** **....................................................................................................**

**Kyoto_F_(A2)**  **............................E...............................................................V.......**

**Loch_Maree_(A3)** **...........T................A.............L..K......................N.TN.....WQKK.SRDA.DNLQN..RI..E.**

**657Ba_(A4)**  **..............Q....A........E.........T..............A.....L...I....N.........KK..S.................**

**H04402065_(A5)**  **...................................................................E................................**

**CDC41370_(A6)**  **....................................................................................................**

**2008-148_(A7)**  **..............I............................................................................EV..I....**

**Chemnitz_(A8)**  **............................E........................................N..............................**

310 320 330 340 350 360 370 380 390 400

....|....|....|....|....|....|....|....|....|....|....|....|....|....|....|....|....|....|....|....|

**ATCC_3502_(A1)**  **KSIVGTTASLQYMKNVFKEKYLLSEDTSGKFSVDKLKFDKLYKMLTEIYTEDNFVKFFKVLNRKTYLNFDKAVFKINIVPKVNYTIYDGFNLRNTNLAAN**

**NCTC2916_(A1(B)** **....................................................................................................**

**Kyoto_F_(A2)**  **...I...................................................N....I.............R.....DE....K.....KGA..ST.**

**Loch_Maree_(A3)** **.T.....TP......I.IR..F....A...I..N.AA.KEF.RV..RGF..LE..NP...I.............R.....DE....NE....E----G..**

**657Ba_(A4)**  **..........................AT...L..R....E...L....................................D.....H.............**

**H04402065_(A5)**  **................................................................................E...................**

**CDC41370_(A6)**  **....................................................................................................**

**2008-148_(A7)**  **...I................................R......................................M....E...................**

**Chemnitz_(A8)**  **................................................................................DE....K.....K.......**

410 420 430 440 450 460 470 480 490 500

....|....|....|....|....|....|....|....|....|....|....|....|....|....|....|....|....|....|....|....|

**ATCC_3502_(A1)**  **FNGQNTEINNMNFTKLKNFTGLFEFYKLLCVRGIITSKTKSLDKGYNKALNDLCIKVNNWDLFFSPSEDNFTNDLNKGEEITSDTNIEAAEENISLDLIQ**

**NCTC2916_(A1(B)** **....................................................................................................**

**Kyoto_F_(A2)**  **.........SR...R....................PF......E...............................D.V....A.................**

**Loch_Maree_(A3)** **S........SR...R....................PF......E...............................D.V....A............S....**

**657Ba_(A4)**  **.....I....K..D.............................E.......E.......................D.V......................**

**H04402065_(A5)**  **...........................................E........................................................**

**CDC41370_(A6)**  **.............A......................................................................................**

**2008-148_(A7)**  **...........................................E...................................................S....**

**Chemnitz_(A8)**  **.........SR........................PF......E...............................D.V......................**

510 520 530 540 550 560 570 580 590 600

....|....|....|....|....|....|....|....|....|....|....|....|....|....|....|....|....|....|....|....|

**ATCC_3502_(A1)**  **QYYLTFNFDNEPENISIENLSSDIIGQLELMPNIERFPNGKKYELDKYTMFHYLRAQEFEHGKSRIALTNSVNEALLNPSRVYTFFSSDYVKKVNKATEA**

**NCTC2916_(A1(B)** **....................................................................................................**

**Kyoto_F_(A2)**  **......D......................P................................D...I....AE....K.NVA......K....I...V..**

**Loch_Maree_(A3)** **......D......................P................................D...I....AE....K.NVA......K....I...V..**

**657Ba_(A4)**  **....N.........T..............P...............N.............K.SN...I....AK....K.NI.......K.I.AI...V..**

**H04402065_(A5)**  **..................................................................V.............S..........R........**

**CDC41370_(A6)**  **......................................................S...........D.............H...................**

**2008-148_(A7)**  **............................................................Y.N...V.I...........S............A.E....**

**Chemnitz_(A8)**  **......D......................P...............................S......................................**

610 620 630 640 650 660 670 680 690 700

....|....|....|....|....|....|....|....|....|....|....|....|....|....|....|....|....|....|....|....|

**ATCC_3502_(A1)**  **AMFLGWVEQLVYDFTDETSEVSTTDKIADITIIIPYIGPALNIGNMLYKDDFVGALIFSGAVILLEFIPEIAIPVLGTFALVSYIANKVLTVQTIDNALS**

**NCTC2916_(A1(B)** **....................................................................................................**

**Kyoto_F_(A2)**  **F...N.A.E.........N..T.M.........V.............S.GE..E.I..T.V.AM......Y.L..F....I..............N....**

**Loch_Maree_(A3)** **VI..S.A.E.........N..T.M.........V............VS.GE..E.IL.T.V.A.......YSL..F....I..............N....**

**657Ba_(A4)**  **VT.VN.I.N.........N....M........V.............I..GE..E.I..........IV....L...........VS..............**

**H04402065_(A5)**  **....................................................................................................**

**CDC41370_(A6)**  **................................................................................I..............N....**

**2008-148_(A7)**  **.......................M.........V............V..KK.EE.............V...VL.I.........TS......R.......**

**Chemnitz_(A8)**  **....................................................................................................**

710 720 730 740 750 760 770 780 790 800

....|....|....|....|....|....|....|....|....|....|....|....|....|....|....|....|....|....|....|....|

**ATCC_3502_(A1)**  **KRNEKWDEVYKYIVTNWLAKVNTQIDLIRKKMKEALENQAEATKAIINYQYNQYTEEEKNNINFNIDDLSSKLNESINKAMININKFLNQCSVSYLMNSM**

**NCTC2916_(A1(B)** **....................................................................................................**

**Kyoto_F_(A2)**  **............T................E...K............................................S.........D...........**

**Loch_Maree_(A3)** **............T................E...K.........R..............................R...R.........D...........**

**657Ba_(A4)**  **...................I.....N...E...K............................................S.........D...........**

**H04402065_(A5)**  **......G...........................................................G.......D.........................**

**CDC41370_(A6)**  **............T................E...K............................................S.........D...........**

**2008-148_(A7)**  **......E..................N........................................G.......D.............D...........**

**Chemnitz_(A8)**  **...........................V..................................................S..T......D...........**

810 820 830 840 850 860 870 880 890 900

....|....|....|....|....|....|....|....|....|....|....|....|....|....|....|....|....|....|....|....|

**ATCC_3502_(A1)**  **IPYGVKRLEDFDASLKDALLKYIYDNRGTLIGQVDRLKDKVNNTLSTDIPFQLSKYVDNQRLLSTFTEYIKNIINTSILNLRYESNH-LIDLSRYASKIN**

**NCTC2916_(A1(B)** **...................................................................................... -............**

**Kyoto_F_(A2)**  **...A....K.....VR.V............VL.......E......A............KK............V.....SIV.KKDD-.......GA...**

**Loch_Maree_(A3)** **...A....K.....VR.V.............L.......E......A..........NDKK............V.....SIV.KKDD-.......GA...**

**657Ba_(A4)**  **...A....K.....VR.V................N...........A............KK............T.A...SIV.KDDD-.......GAE.Y**

**H04402065_(A5)**  **...................................................................................... -.........E..**

**CDC41370_(A6)**  **...A....K.....VR.V.............................................................S....N. -............**

**2008-148_(A7)**  **..Q...Q.K...T..R.S......................................A............................. -............**

**Chemnitz_(A8)**  **...A....K.....VREV.............L..............A............KK............T.....SIVVDKDGR.......GAE.Y**

910 920 930 940 950 960 970 980 990 1000

....|....|....|....|....|....|....|....|....|....|....|....|....|....|....|....|....|....|....|....|

**ATCC_3502_(A1)**  **IGSKVNFDPIDKNQIQLFNLESSKIEVILKNAIVYNSMYENFSTSFWIRIPKYFNSISLNNEYTIINCMENNSGWKVSLNYGEIIWTLQDTQEIKQRVVF**

**NCTC2916_(A1(B)** **....................................................................................................**

**Kyoto_F_(A2)**  **..DR.YY.S......K.I.....T........................K.....SK.N..........I.....................NKQNI.....**

**Loch_Maree_(A3)** **..DR.YY.S......K.I.....T........................K.....SK.N..........I.....................NKQNI.....**

**657Ba_(A4)**  **N.D..YYNS......R.I.....T......K.....................................................................**

**H04402065_(A5)**  **..........................I.....................K.....SK.N..........I.....................NKQNI.....**

**CDC41370_(A6)**  **...R............................................K.....SE............I.....................NKQNI.....**

**2008-148_(A7)**  **...R............................................K.....SK.N..........I.....................NEQNI.....**

**Chemnitz_(A8)**  **N.D..SYNS......K.I.....A........................K.....SK.N..........I.....................N.QNI.....**

1010 1020 1030 1040 1050 1060 1070 1080 1090 1100

....|....|....|....|....|....|....|....|....|....|....|....|....|....|....|....|....|....|....|....|

**ATCC_3502_(A1)**  **KYSQMINISDYINRWIFVTITNNRLNNSKIYINGRLIDQKPISNLGNIHASNNIMFKLDGCRDTHRYIWIKYFNLFDKELNEKEIKDLYDNQSNSGILKD**

**NCTC2916_(A1(B)** **....................................................................................................**

**Kyoto_F_(A2)**  **.....V...................TK.........................K..........PR...M.....................S.........**

**Loch_Maree_(A3)** **.....V.........M.........TK.........................K..........PR...M.....................S...P.....**

**657Ba_(A4)**  **........................ITK.........................K..........P....V...........S...................**

**H04402065_(A5)**  **.....VA..........I.............................................P....................................**

**CDC41370_(A6)**  **.....VA..........I.......TK.........................K..........PR...M.....................S.........**

**2008-148_(A7)**  **.....V...................TK.........................K..........P....L...............................**

**Chemnitz_(A8)**  **.....V...................DK....................................PR...V...............................**

1110 1120 1130 1140 1150 1160 1170 1180 1190 1200

....|....|....|....|....|....|....|....|....|....|....|....|....|....|....|....|....|....|....|....|

**ATCC_3502_(A1)**  **FWGDYLQYDKPYYMLNLYDPNKYVDVNNVGIRGYMYLKGPRGSVMTTNIYLNSSLYRGTKFIIKKYASGNKDNIVRNNDRVYINVVVKNKEYRLATNASQ**

**NCTC2916_(A1(B)** **....................................................................................................**

**Kyoto_F_(A2)**  **...N.............F..........I...............V........T..E.............E.............................**

**Loch_Maree_(A3)** **...N.............F..........I........................T..M.............E.............................**

**657Ba_(A4)**  **..........S..............................DN.............M...........................................**

**H04402065_(A5)**  **...N.......................................IV...........M...........................................**

**CDC41370_(A6)**  **...N.............F.....................S.STLL........G..M..............................N............**

**2008-148_(A7)**  **.......................I....I...............T........M..M........H...................L..............**

**Chemnitz_(A8)**  **............................I...............V........T..M.........................................L.**

1210 1220 1230 1240 1250 1260 1270 1280 1290

....|....|....|....|....|....|....|....|....|....|....|....|....|....|....|....|....|....|....|..

**ATCC_3502_(A1)**  **AGVEKILSALEIPDVGNLSQVVVMKSKNDQGITNKCKMNLQDNNGNDIGFIGFHQFNNIAKLVASNWYNRQIERSSRTLGCSWEFIPVDDGWGERPL**

**NCTC2916_(A1(B)** **.................................................................................................**

**Kyoto_F_(A2)**  **...........................D....R.....................LYD..............VGKA...F...............SS.**

**Loch_Maree_(A3)** **...........................D....R.................V...LYD..............VGKA...F...............SS.**

**657Ba_(A4)**  **............................................................................................R....**

**H04402065_(A5)**  **........V.......................R..........................D..................F...............S..**

**CDC41370_(A6)**  **..............I.................R.....................K..D.Y.............I....F...............K..**

**2008-148_(A7)**  **..G......V......................R.......................................GKT.V.......L....Y....SS.**

**Chemnitz_(A8)**  **................................R................L.....................VGKA...F...............SSQ**

Light chain: dark grey; heavy chain: light grey; unique amino acid differences in BoNT/A8 based on the representatives of BoNT/A1 to A7 are marked in red; arginine insertion in position 888 is marked in green; amino acids important for catalytic activity are marked in magenta; essential amino acids for ganglioside-binding (E…H…SXWY..G) motif: turquoise.
