# Supplementary material for: Isolation and Functional Characterization of the Novel Clostridium botulinum Neurotoxin A8 Subtype
Source: PLoS One. 2015 Feb 6;10(2):e0116381. doi: 10.1371/journal.pone.0116381 (PMC4320087; doi:10.1371/journal.pone.0116381)
Supplement: S2 Fig — Amino acid coverage of BoNT/A8 as observed after combined endopeptidase digest and LC-MS/MS is shown in amber; unique amino acid differences present in BoNT/A8 based on the representatives of BoNT/A1 to A7 are marked in red; mutations at positions also found in other BoNT/A subtypes are marked in yellow (see also Fig. 2); arginine insertion at position 888 is marked in green. (DOCX) [file pone.0116381.s002.docx]

**Figure S2:** Amino acid sequence coverage after combined endopeptidase digest and tandem mass spectrometry analysis

**1** MPFVNK**QFNY KDTVNGIDIA YIKIPNAGQM QPVK**AFKIHN K**IWVIPERDT**

**51 FTNPK**EGDLN PPPEAK**QVPV SYYDSTYLST DNEKDNYLK**G VTKLFERIYS

**101** TDLGR**MLLTS IVRGIPFWGG STIDTELKVI DTNCINVIQP DGSYR**SEELN

**151** LVIIGPSADI IQFECK**SFGH DVLNLTRNGY GSTQYIR**FSP DFTFGFEESL

**201** EVDTNPLLGA GK**FATDPAVT LAHELIHAEH RLYGIAINPN R**VFK**VNTNAY**

**251 YEMSGLEVSF EELR**TFGGHN AK**FIDSLQEN EFR**LYYYNK**F KDIASTLNK**A

**301** K**SIVGTTASL QYMK**NVFKEK **YLLSEDTSGK** FSVDKLKFDK LYK**MLTEIYT**

**351 EDNFVK**FFKV LNRK**TYLNFD K**AVFK**INIVP DENYTIKDGF NLKNTNLAAN**

**401 FNGQNTEINS RNFTKLKNFT GLFEFYK**LLC VRGIIPFKTK SLDEGYNKAL

**451** NDLCIK**VNNW DLFFSPSEDN FTNDLDK**VEE ITSDTNIEAA EENISLDLIQ

**501** QYYLTFDFDN EPENISIENL SSDIIGQLEP MPNIERFPNG K**KYELDKYTM**

**551 FHYLR**AQEFE HSKSR**IALTN SVNEALLNPS RVYTFFSSDY VK**K**VNKATEA**

**601 AMFLGWVEQL VYDFTDETSE VSTTDKIADI TIIIPYIGPA LNIGNMLYK**D

**651** DFVGALIFSG AVILLEFIPE IAIPVLGTFA LVSYIANK**VL TVQTIDNALS**

**701 K**R**NEKWDEVY KYIVTNWLAK VNTQIDLVR**K **KMKEALENQA EATKAIINYQ**

**751 YNQYTEEEKN NINFNIDDLS SKLNESINSA MTNINKFLDQ CSVSYLMNSM**

**801 IPYAVKR**LKD FDASVREVLL KYIYDNR**GTL ILQVDRLKDK VNNTLSADIP**

**851 FQLSK**YVDNK **KLLSTFTEYI KNITNTSILS IVVDKDGRLI DLSRYGAEIY**

**901 NGDKVSYNSI DKNQIKLINL ESSAIEVILK** NAIVYNSMYE NFSTSFWIKI

**951** PKYFSKINLN NEYTIINCIE NNSGWK**VSLN YGEIIWTLQD NQQNIQR**VVF

**1001** K**YSQMVNISD YINRWIFVTI TNNRLDK**SKI YINGR**LIDQK PISNLGNIHA**

**1051 SNNIMFK**LDG CRDPRRYIVI K**YFNLFDKEL NEKEIKDLYD NQSNSGILKD**

**1101 FWGDYLQYDK PYYMLNLYDP NKYVDVNNIG IRGYMYLKGP RGSVVTTNIY**

**1151 LNSTLYMGTK** FIIKK**YASGN KDNIVRNNDR VYINVVVKNK** EYR**LATNALQ**

**1201 AGVEKILSAL EIPDVGNLSQ VVVMK**SK**NDQ GIRNK**CK**MNL QDNNGNDIGL**

**1251 IGFHQFNNIA KLVASNWYNR** QVGKASRTFG CSWEFIPVDD GWGESSQ

Amino acid coverage of BoNT/A8 as observed after combined endopeptidase digest and LC-MS/MS is shown in amber; unique amino acid differences present in BoNT/A8 based on the representatives of BoNT/A1 to A7 are marked in red; mutations at positions also found in other BoNT/A subtypes are marked in yellow (see also Fig. 2); arginine insertion at position 888 is marked in green.
